# The effect of cholesteryl ester transfer protein on pancreatic beta cell dysfunction in mice

**DOI:** 10.1186/s12986-016-0082-1

**Published:** 2016-03-11

**Authors:** Wen Guo, Yingyun Gong, Zhenzhen Fu, Jinxiang Fu, Yan Sun, Xianxia Ju, Yina Chang, Wen Wang, Xiaohui Zhu, Beibei Gao, Xiaoyun Liu, Tao Yang, Hongwen Zhou

**Affiliations:** Department of Endocrinology, the First Affiliated Hospital of Nanjing Medical University, 300 Guangzhou Road, Nanjing, 210029 China

**Keywords:** Cholesterol ester transfer protein, Free cholesterol, Beta cell function, Glucose metabolism

## Abstract

**Background:**

Cholesterol accumulation causes pancreatic beta cell lipotoxicity and dysfunction. Cholesteryl ester transfer protein (CETP) plays an important role in blood lipid homeostasis. However, its role in tissue lipid metabolism remains unclear. We hypothesized that plasma CETP impact cholesterol homeostasis in the beta cells, thus damaging their functions.

**Methods:**

The adipose tissue-specific CETP expression transgenic (aP2-CETPTg) mice, characterized by high CETP levels in the circulation, were used in this study. Pancreatic islet cholesterol and beta cell function were assessed in mice. We further measured mRNA levels of the genes involved in beta cell proliferation and differentiation, inflammation and cholesterol metabolism. TUNEL assay was applied to investigate beta cell apoptosis in islets.

**Results:**

The aP2-CETPTg mice exhibited glucose intolerance, lower plasma insulin concentrations but increased insulin sensitivity compared with wild type mice. In addition, glucose-stimulated insulin secretion from isolated pancreatic islets significantly decreased, and free cholesterol significantly increased. Moreover, the number and size of islets from aP2-CETPTg mice were significantly decreased. Genes involved in beta cell proliferation, such as Pdx1 and BETA2, were down-regulated; genes involved in inflammation and ER stress, such as IL-1β, CHOP, and Xbp1 were up-regulated, in line with an increase of beta cell apoptosis.

**Conclusions:**

Plasma CETP cause**s** free cholesterol accumulation in islets which could contribute to beta cell dysfunction. Thus, CETP inhibition could be a novel protective strategy for dyslipidemia related to diabetes and obese.

## Background

The increased morbidity, mortality of type 2 diabetes mellitus (T2DM) makes it a crucial public health condition. Islet beta cell dysfunction and insulin resistance are major characteristics of pathophysiology of T2DM. Although insulin resistance occurs earlier during disease onset, beta cell dysfunction predates long before the occurrence of clinical hyperglycemia [1]. Many potential mechanisms for islet beta cell dysfunction have been proposed [2], however it still remains unclear. Lipotoxicity is regarded as one of the possible mechanisms which contribute to beta cell dysfunction. It has been suggested that increased free fatty acids accumulation in beta cells could reduce insulin secretion from beta cell, and induce beta cell apoptosis [3]. Recent studies have indicated that the accumulation of free cholesterol in beta cells may also contribute to lipotoxicity [4] and beta cell dysfunction [5].

Cholesteryl ester transfer protein (CETP) is a hydrophobic plasma glycoprotein that mediates the cholesteryl ester (CE) transfer from high density lipoprotein (HDL) to very low density lipoprotein (VLDL) or low density lipoprotein (LDL) in exchange for triglycerides, leading to lower HDL concentrations and more small dense-LDL particles [6]. In human, CETP is highly expressed in adipose tissue and liver, where it can be secreted into the blood and then participates in cholesterol metabolism [7]. We speculated that circulating CETP may mediate cholesterol homeostasis in islet beta cells. Some studies indicate that CETP may have additional effects on glucose metabolism other than lipid metabolism. A sub-study of the Investigation of Lipid Level Management to Understand Its Impact in Atherosclerotic Events (ILLUMINATE) trial reported that treatment with CETP inhibitor (torcetrapib) improved glycemic control in atorvastatin-treated patients with T2DM [8]. Siebel et al. [9] found that CETP inhibitor increased postprandial insulin and promoted ex vivo beta cell glucose-stimulated insulin secretion, potentially via enhanced beta cell cholesterol efflux. Most clinical studies have shown that CETP mass was increased in patients with T2DM and obesity [10]. CETP activity was positively correlated with fasting blood glucose and HbA1c while negatively correlated with HDL-C in T2DM [11]. Few studies also reported diabetes may suppress hepatic CETP in obese [12, 13]. Our previous study showed that glucose uptake and intracellular cholesterol were increased when 3T3-L1 adipocyte was transfected with CETP [14]. All these studies indicated that CETP might play a role in glucose metabolism.

We previously generated adipose tissue-specific CETP expression transgenic (aP2-CETPTg) mice and demonstrated that this model have the following characteristics: 1) high circulating CETP secreted by adipose tissue; 2) elevated LDL and declined HDL levels compared with WT mice; 3) very low CETP expression in other tissues (including skeletal muscle, liver, lung, kidney, heart, and brain) [15]. Natural flanking region (NFR)-CETPTg mice is another CETP transgenic mouse model with human like CETP concentration in the circulation and tissue distributions (liver, spleen, small intestine, kidney, and adipose tissue) [16]. Recently, Raposo et al. reported physiological levels of CETP (~2 μg/ml) did not affect glucose homeostasis and insulin secretion [17]. Interestingly, we previously found that over-expressed CETP might modulate glucose metabolism and insulin action in addition to its effects on lipoprotein metabolism in 3T3-L1 adipocytes [14]. In order to investigate whether circulating CETP (other than local CETP expression) and elevated CETP activity play roles in regulation of glucose metabolism and beta cell lipid accumulation, we used aP2-CETPTg mouse to explore the effects of CETP on mouse pancreatic beta cell lipid composition and beta cell function.

## Methods

### Animals

The NFR-CETPTg and aP2-CETPTg mice have C57BL/6J background [15]. Transgenic mice and non-transgenic littermates were fed standard rodent chow diet, housed in cages in a pathogen-free facility, and maintained on a 12 h light/12 h dark cycle. All experiments were performed on 10~16 week-old male mice. All animal experiments were conducted in accord with accepted standards of humane animal care, as outlined in the Ethical Guidelines, and were approved by the Research Animal Care Committee of Nanjing Medical University (IACUC-14030136).

### CETP activity measurement

Mouse plasma CETP activity was measured by a fluorescence method as previously described [15]. Briefly, NBD-CE works as the donor, and ultracentrifugation separated human VLDL/LDL works as the acceptor. Reactions were performed in a 100 μl reaction mixture containing 3 μl plasma sample. Finally, fluorescence were measured by fluorescence microplate (SynergyMx, BioTek, United States) under 460 nm excitation wave and 530 nm emission wave. Plasma CETP activity was presented as detected fluorescence value (arbitrary units)/3 μl plasma).

### Glucose and insulin tolerance test

Intraperitoneal glucose tolerance test (IPGTT) was performed on 16 h-fasted mice injected with D-glucose at 1 g/kg body weight. Blood glucose levels were measured at 0, 15, 30, 60 and 120 min after glucose injection. Plasma insulin during IPGTT was measured by Mouse Insulin ELISA kit (Millipore-linco, Billerica, MA, USA). Intraperitoneal insulin tolerance test (IPITT) was performed on 5 h-fasted mice injected with recombinant human insulin (Humulin, Eli Lilly and company, China) at 0.75 mU/kg body weight. Blood glucose levels were measured at 0, 15, 30, 60 and 120 min after insulin injection.

### Isolation of pancreatic islets

Primary mouse islets were isolated by collagenase V (Sigma-Aldrich Chemie, Steinheim, Germany) digestion and filtration as previously described [18]. Islets used for glucose-stimulated insulin secretion measurement were cultured in RPMI-1640 (Gibco, California, USA) supplemented with 10 % FCS (Gibco, California, USA), 100 U/ml penicillin and 100 μg/ml streptomycin. Islets used for cholesterol measurement and real-time PCR were washed with PBS and frozen down immediately after isolation. Isolated islets number counting was conducted by two persons in a double-blinded fashion, referring to the method used by Ishikawa [19].

### Glucose-stimulated insulin secretion assay

After overnight culture, islets were washed and used for glucose-stimulated insulin secretion (GSIS). Islets (eight similar-sized islets per well and eight wells per group) were firstly pre-incubated in Krebs-Ringer bicarbonate HEPES buffer supplemented with 0.2 % BSA for 1 h at 37 °C and 5 % CO_2_, then incubated with KRB/HEPES buffer supplemented with 0.2 % BSA containing 3.3 or 16.7 mmol/l glucose for 1 h [18]. After then, islets were incubated with acid-ethanol (1.5 % HCl in 70 % Ethanol) overnight for insulin content measurement. The supernatants were collected after each treatment. Insulin levels were measured by the radioimmunoassay kit (BNIBT, Beijing, China). Insulin secretion ability is calculated by the relative secreted insulin to insulin content.

### Plasma lipid measurement

Overnight fasting plasma were collected for plasma lipid measurement. The total cholesterol (TC), free cholesterol (FC) were assayed by enzymatic methods (Applygen Technologies Inc. Beijing, China), according to the instructions provided by the manufacturers. CE concentration is calculated by TC minus FC.

### Islet lipid determination

Twenty similar-sized islets were washed five times with PBS and then cellular lipids were extracted by hexane: isopropyl alcohol (2:1 *v/v*) as described [20], using β-sitosterol (5 μg/ml) as an internal standard. Samples were split into two equal fractions to measure FC and TC separately. For TC measurement, CE was firstly transformed into free cholesterol. Finally, CE content is calculated by TC minus FC. To hydrolyze cellular cholesteryl esters prior to gas chromatography, 50 % KOH (200 μl) and methanol (3 ml) were added to dried aliquots and heated to 80 °C for 1 h in argon-capped tubes. After cooling, 3 ml H_2_O and 5 ml HPLC grade hexane were added. Samples were centrifuged at 1000 rpm for 5 min and supernatant containing cholesterol was removed and dried under nitrogen. Cholesterol mass was measured by injection of 1 μl aliquots into a 5890 Series II gas chromatograph with a Detector (FID DETECTOR) using a 15 m × 0.53 mm × 1.5 μm cross-linked HP-5 Trace Analysis (5 % Ph Me Siloxane) column. Gas chromatography was performed at an isothermal 260 °C, injection port temperature of 300 °C, and detector temperature of 300 °C with nitrogen carrier flow rate of 30 ml/min. Calibration was achieved by injection of a standard solution of cholesterol (100 μg/ml).

### RNA extraction, reverse transcription and gene expression

Total RNA from isolated islets were extracted using the RNAiso Plus (Takara, Dalian, China) and reverse-transcribed into cDNA using the Prime Script RT reagent kit (Takara, Dalian, China). Data for triplex real-time PCR assay were collected with an AB 7500 Real time PCR system (Applied Biosystems, Inc. United States). Reactions were performed with SYBR Green PCR Master Mix (Takara, Dalian, China) under following thermal cycling conditions: 2 min at 50 °C, 10 min at 95 °C, followed by 40 cycles of 95 °C for 15 s and 60 °C for 1 min. Conventional PCR reactions were performed in a 20 μl reaction mixture containing 0.5 μmol/L primers and 12.5 mg/L cDNA. Relative gene expression data were analyzed in the 2-ΔΔCT method, and the results were expressed as the expression fold over the control group. In exception, since there is no CETP mRNA expression in WT mice, the relative CETP mRNA of adipose tissue from aP2-CETPTg mice and NFR-CETPTg mice and of islets from NFR-CETPTg mice were expressed as 2^-ΔCT. The following primer sequences were used:

CETP310-A, 5′-TAGTGTTTACAGCCCTCATGAACAGCAAAG- 3′;

CETP310-B, 5′-CTCCATCTCCGTACTCCTAACCCAACTTCC- 3′.

Ki-67 forward, 5′-ATCATTGACCGCTCCTTTAGGT- 3′;

Ki-67 reverse, 5′-GCTCGCCTTGATGGTTCCT- 3′.

Pdx1 forward, 5′-GATGAAATCCACCAAAGCTCA- 3′;

Pdx1 reverse, 5′-AGAATTCCTTCTCCAGCTCCA- 3′.

BETA2 forward, 5′-ACTCCAAGACCCAGAAACTGTC- 3′;

BETA2 reverse, 5′-ACTGGTAGGAGTAGGGATGCAC- 3′.

IL-1β forward, 5′- TGCAGAGTTCCCCAACTGGTACATC- 3′;

IL-1β reverse, 5′- GTGCTGCCTAATGTCCCCTTGAATC- 3.

TNF-α forward, 5′- CCCTCACACTCAGATCATCTTCT- 3′;

TNF-α reverse, 5′- GCTACGACGTGGGCTACAG- 3′.

IL-6 forward, 5′- CCAAGAGGTGAGTGCTTCCC- 3′;

IL-6 reverse, 5′- CTGTTGTTCAGACTCTCTCCCT- 3′.

IL-10 forward, 5′- GCTCTTACTGACTGGCATGAG- 3′;

IL-10 reverse, 5′- CGCAGCTCTAGGAGCATGTG- 3′.

CHOP forward, 5′-CATACACCACCACACCTGAAAG- 3′;

CHOP reverse, 5′-CCGTTTCCTAGTTCTTCCTTGC- 3′.

Atf6 forward, 5′- GACTCACCCATCCGAGTTGTG- 3′;

Atf6 reverse, 5′- CTCCCAGTCTTCATCTGGTCC- 3′.

Xbp1 forward, 5′- AGCAGCAAGTGGTGGATTTG- 3′;

Xbp1 reverse, 5′- GAGTTTTCTCCCGTAAAAGCTGA- 3′.

Ldlr forward, 5′-CTGTTCCCA CCTCTGTTT AC- 3′;

Ldlr reverse, 5′- AGTGAG ATACGGCGA ATA GA- 3′.

HMGCS forward, 5′-TGG GAC CAA CCT TCT ACC TC- 3′;

HMGCS reverse, 5′-CAT CAA GGA CAG CTC ACC AG- 3′.

Srebp2 forward, 5′-TAACCCCTTGACTTCCTTGCT- 3′;

Srebp2 reverse, 5′-TGCTCTTAGCCTCATCCTCCA- 3′.

### Histology and immunohistochemistry

Pancreata were isolated, fixed with 10 % buffered formalin, embedded in paraffin, sectioned and stained with hematoxylin and eosin for histological analysis. Pancreatic sections were scanned by Zeiss microscope at ×200 magnifications and islet size was analyzed with AxioVision software. For analysis of pancreatic islet apoptosis, double staining of in situ cell death detection fluorescence and insulin fluorescent immunohistochemistry were performed. The following primary antibodies were used: Rabbit anti-insulin polyclonal antibody, 1:50 (Proteintech Group, Inc. Chicago, USA); Secondary antibodies DyLight594 rabbit anti-goat IgG[H+L], 1:300 (MultiSciences Biotech, Hangzhou, China). The beta cell apoptosis was assessed by transferase-mediated dUTP nick-end labeling (TUNEL) assay (keyGEN, Nanjing, China) according to the manufacturer’s protocol. For the determination of the overall rate of apoptosis, each section stained for insulin, TUNEL, and DAPI were imaged at 400 magnification (40 × objective), and the total number of TUNEL-positive cells per field were quantified. To determine the frequency of beta cell apoptosis, the number of cells positive for TUNEL was quantified in each islet and expressed as percentage of the total number of beta cells (*n* = ~4000). Beta cells were counted in 16 week old of age male mice per genotype (*n* = 4–5).

### Statistical analysis and calculation

Data are expressed as means ± SD. Differences between groups were calculated by Student’s *t* test. Data were analyzed using GraphPad Prism Software version 5.0, with significance defined as *p* < 0.05 (two-tailed). HOMA-IR is calculated by fasting plasma glucose (mmol/L) × fasting insulin (mIU/L)/22.5; HOMA-β is calculated by 20 × fasting insulin (mIU/L)/(fasting plasma glucose (mmol/L) − 3.5) (%).

## Results

### Characteristics of CETP transgenic mice

**Fig. 1:**
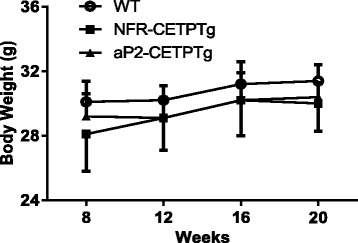
Body weight of NFR-CETPTg, aP2-CETPTg and WT mice. Body weight records at 8, 12, 16 and 20 week-old mice. No significant difference between NFR-CETPTg, aP2-CETPTg and WT mice (*n* = 6 per group). Values are expressed as mean ± SD

**Fig. 2:**
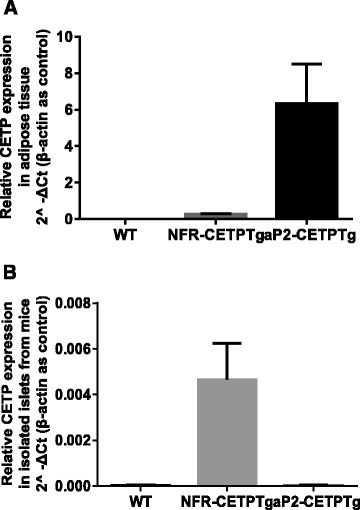
CETP mRNA expression in adipose tissue and islets. Real-time PCR CETP mRNA analysis in mouse adipose tissue (**a**) and isolated islets (**b**). In aP2-CETPTg mice, CETP is predominantly expressed in adipose tissue but not detectable in islets, while a regular CETP expression in NFR-CETPTg mice. No CETP gene expression is noted in their controls. *n* = 6 per group. Values are expressed as mean ± SD

Wild-type (WT), NFR-CETPTg and aP2-CETPTg mice did not show differences in body weight in the age of 8 to 20 week (Fig. 1). The CETP mRNA in the adipose tissue was significantly higher in aP2-CETPTg mice than in NFR-CETPTg mice, but not expressed in WT littermates as expected (Fig. 2a). In consistent with the real-time PCR results, no CETP activity was detected in WT mice whereas high plasma CETP levels in aP2-CETPTg mice (OD value = 12,600 ± 784/μl), which is up to 2.3 folds of that in human plasma (OD value = 5570 ± 171/μl). Moreover, there is no detectable CETP mRNA expression in the isolated islets from WT and aP2-CETPTg mice, while a certain CETP expression in the islets from NFR-CETPTg mice (Fig. 2b).

### aP2-CETPTg mice exhibit impaired glucose tolerance and insulin secretion

**Fig. 3:**
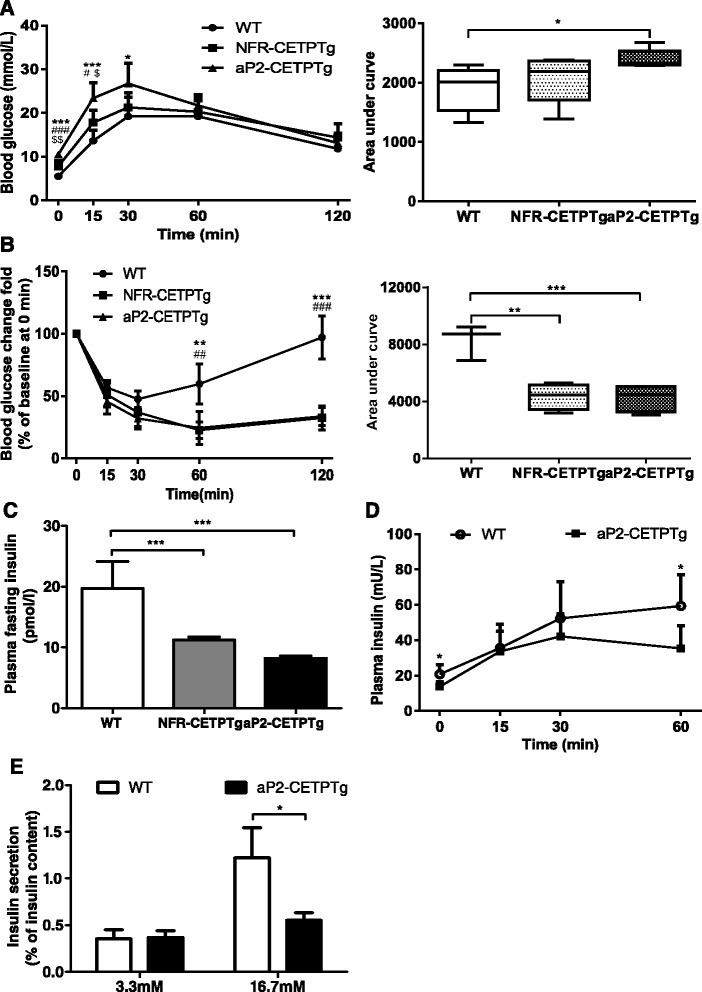
Impaired glucose tolerance and decreased insulin secretion in aP2-CETPTg mice in vivo and in vitro. **a** IPGTT was performed in 10–12 week-old mice by intraperitoneal injecting D-glucose (1 g/kg body weight). Blood glucose levels were measured at 0, 15, 30, 60 and 120 min during IPGTT. *n* = 6 per group. Area under curve is calculated and showed (*right*). The aP2-CETPTg mice have impaired glucose tolerance than WT mice. **b** IPITT was performed in 10–12 week-old mice by intraperitoneal injecting insulin (0.75 IU/kg body weight). Blood glucose were recorded at 0, 15, 30, 60 and 120 min during IPITT. *n* = 6 per group. Area under curve is calculated and showed (*right*). The insulin sensitivity is increased in the NFR-CETPTg and aP2-CETPTg mice. **c** Plasma fasting insulin levels in NFR-CETPTg, aP2-CETPTg and WT mice. CETP transgenic mice exhibited decreased fasting insulin secretion than WT mice. *n* = 5 per group. **d** Plasma insulin levels were measured at 0, 15, 30 and 60 min during IPGTT in WT and aP2-CETPTg mice. After glucose challenge, aP2-CETPTg mice secreted less insulin than WT mice. *n* = 6 per group. **e** Insulin secretion ability was measured from isolated islets by glucose-stimulated insulin secretion. Eight islets per well, eight wells per group. Insulin release in response to 16.7 mmol/l glucose was significantly impaired in aP2-CETPTg mice compared with WT mice. Values are expressed as mean ± SD. *p < 0.05 compared with control group, **p < 0.01 compared with control group. *IPGTT* intraperitoneal glucose tolerance test, *IPITT* intraperitoneal insulin tolerance test

To study the effects of CETP on systemic glucose metabolism and insulin secretion in NFR-CETPTg and aP2-CETPTg mice, we conducted IPGTT, IPITT in vivo. A glucose challenge (1 g/kg) revealed an impaired glucose tolerance in aP2-CETPTg mice (*p* < 0.05), compared with WT mice, while a glucose intolerance tendency in NFR-CETPTg mice (Fig. 3a). Interestingly, we also found that NFR-CETPTg and aP2-CETPTg mice had an increased sensitivity to insulin compared with WT mice (Fig. 3b). The homeostasis model assessment of insulin resistance (HOMA-IR) of NFR-CETPTg and aP2-CETPTg mice were lower compared with WT mice (4.24 ± 1.19, 2.47 ± 0.54, 6.14 ± 3.14, respectively, *p* < 0.05), which also suggested that insulin sensitivity of aP2-CETPTg mice were higher. Besides, both NFR-CETPTg and aP2-CETPTg mice had significantly decreased plasma fasting insulin than WT mice (*p* < 0.001) (Fig. 3c). We further measured insulin levels after glucose overload, and we found that aP2-CETPTg mice had less insulin secretion than WT mice, especially at 0 and 60 min (*p* < 0.05) (Fig. 3d). Homeostasis model assessment of beta cell function index (HOMA-β) of NFR-CETPTg and aP2-CETPTg mice were lower compared with WT mice (48.7 ± 26.2, 56.9 ± 24.0, 96.6 ± 31.6, repectively, *p* < 0.05), which indicated beta cell dysfunction in aP2-CETPTg mice. Furthermore, we found that islets from aP2-CETPTg mice had a significant reduction in glucose-stimulated insulin secretion in vitro compared with that from WT mice (*p* < 0.05) (Fig. 3e). Collectively, both NFR-CETPTg and aP2-CETPTg mice have impaired glucose tolerance and insulin secretion, but increased insulin sensitivity. Moreover, we also found that there was a CETP dose-dependent effect on glucose tolerance.

### aP2-CETPTg mice have fewer and smaller islets

**Fig. 4:**
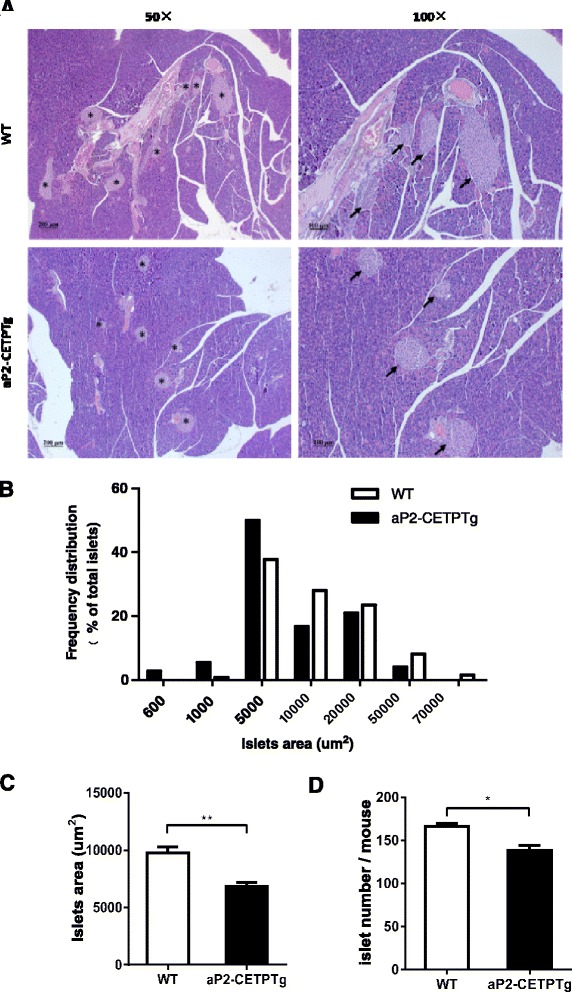
Morphology, number and area distribution of islets from aP2-CETPTg mice. **a** Representative pancreas sections of aP2-CETPTg mice and WT mice were stained with hematoxylin and eosin for histological analysis. Original magnification, ×50 (*left panels*); ×100 (*right panels*). Islets are marked with *asterisk* and *arrow*. **b** The area distribution of islets on H-E staining pancreas sections. (aP2-CETPTg, *n* = 9; WT, *n* = 10; 10 sections at two separate levels per mouse pancreas). Islets from aP2-CETPTg mice were smaller in size than that from WT mice. **c** Area quantification of islets on pancreas sections displayed significant decreased area in aP2-CETPTg mice. **d** Pancreatic islets were isolated from aP2-CETPTg mice and WT mice (*n* = 6 per group) and double-blinded counted by two persons. Islets isolated from aP2-CETPTg mice were fewer than that from WT mice. Values are expressed as mean ± SD. **p < 0.01, compared with control group

Islets from aP2-CETPTg mice exhibited less islet number (138 ± 14 vs.166 ± 10, *p* < 0.05) (Fig. 4d) and smaller size (6850 ± 6940 vs. 9780 ± 10,100 μm^2^, *p* < 0.05) compared with WT mice, which was clearly reflected by frequency distribution and mean area quantification on the pancreatic H-E staining sections (10 sections from two separate levels per mouse, 9–10 mice per group) (Fig. 4a–c).

### aP2-CETPTg mice have higher apoptosis rate in beta cell

**Fig. 5:**
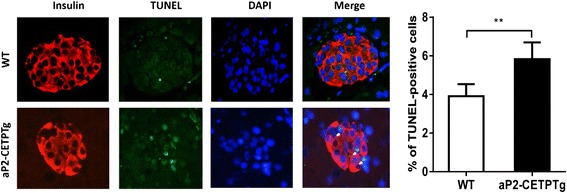
Increased beta cell apoptosis in aP2-CETPTg mice compared with WT mice by TUNEL. Representative TUNEL-positive beta cells were marked with *white arrows* (*Left*). The number of cells positive for TUNEL was quantified in every single islet and expressed as percentage of the total number of beta cells (*n* = ~4000) (*Right*). Beta cells were counted in immunofluorescence staining pancreas section form 16 week-old male mice (*n* = 5 per group). Values are expressed as mean ± SD. **p < 0.01, compared with control group

**Fig. 6:**
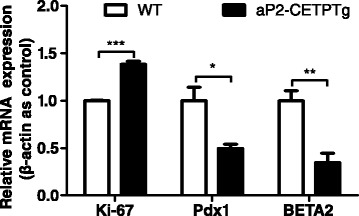
Expression of proliferation and differentiation related genes in isolated islets from aP2-CETPTg mice. Ki-67 mRNA level was significantly upregulated in islets from aP2-CETPTg mice. The mRNA expression of Pdx1 and BETA2 were decreased compared with WT mice. Values are expressed as mean ± SD. *p < 0.05, **p < 0.01, and *** *p* < 0.001 compared with control group. *n* = 5 per group. *Pdx1* pancreatic and duodenal homeobox 1

We next sought to determine whether decreased insulin secretion and smaller sized islets in aP2-CETPTg mice result from increased apoptosis of beta cells? Immunofluorescence stating of pancreas sections were conducted to quantify apoptosis rate of beta cells in aP2-CETP and WT mice. Results showed that the frequency of TUNEL-positive beta cell significantly increased in aP2-CETPTg mice (Fig. 5), indicating that circulating CETP could induce beta cell apoptosis. To further explain the reduction of islet mass, we next measured general proliferation marker Ki-67 and beta cell specific transcription factors, pancreatic and duodenal homeobox 1 (Pdx1) and BETA2. Ki-67 mRNA level was increased but the latter two both were suppressed in aP2-CETPTg mice (Fig. 6).

### Circulating CETP promotes free cholesterol accumulation in the islets

**Fig. 7:**
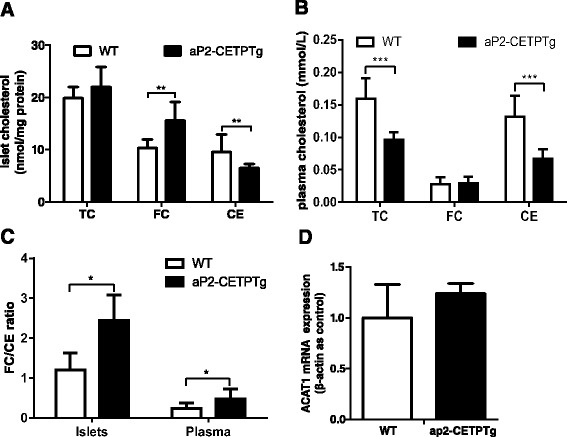
Lipid profile in plasma and islets from ap2-CETPTg mice. **a** Total cholesterol, free cholesterol, and cholesteryl ester of islets isolated from aP2-CETPTg mice and WT mice (20 islets per mice, 5 mice per group) were extracted and measured as described in the Methods. **b** Plasma cholesterol profile in aP2-CETPTg and WT mice (*n* = 10 per group). **c** The FC/CE ratio in plasma and islets. **d** ACAT1 mRNA expression does not differ in the two groups. Values are expressed as mean ± SD. *p < 0.05 and **p < 0.01, ***p < 0.001, compared with control group

**Fig. 8:**
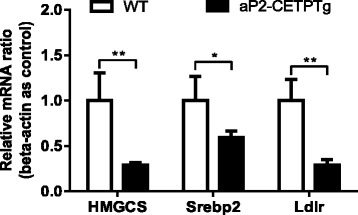
Expression of cholesterol synthesis and uptake related genes in isolated islets from aP2-CETPTg mice. Relative mRNA levels of HMGCS, Srebp2 and Ldlr in isolated islets. *n* = 5 per group. Values are expressed as mean ± SD. *p < 0.05 and **p < 0.01, compared with control group. *HMGCS* 3-hydroxy-3-methylglutaryl-CoA synthase, *Srebp2* sterol regulatory element-binding protein 2, *Ldlr* LDL receptor

To investigate the effect of CETP on islet lipid content, we utilized gas chromatograph to determine the lipid profile in the islets and found that FC (15.6 ± 3.60 vs. 10.3 ± 1.62 mmol/mg protein, *p* < 0.05), FC/CE ratio (2.44 ± 0.640 vs. 1.20 ± 0.430, *p* < 0.05) but not total cholesterol (22.0 ± 3.83 vs. 19.9 ± 2.13 mmol/mg protein, *p* = 0.216) were significantly increased in the transgenic mice islets compared with controls (Fig. 7a). In addition, plasma TC and CE were significantly decreased in aP2-CETPTg mice than WT mice (Fig. 7b), no difference was observed in FC (Fig. 7b) but FC/CE ratio induced (Fig. 7c). ACAT1 converts FC to CE, playing an important role in cellular cholesterol homeostasis, but ACAT1 mRNA in islets did not differ between the two groups (Fig. 7b). We further found that mRNA levels of cholesterol synthesis and uptake related genes, 3-hydroxy-3-methylglutaryl-CoA synthase (HMGCS), sterol regulatory element-binding protein-2 (SREBP-2), and LDL receptor (LDLR) were all significantly reduced (71, 41, 71 %, respectively) (Fig. 8).

### The effect of circulating CETP on the expression of genes participated in islet inflammation and ER stress

**Fig. 9:**
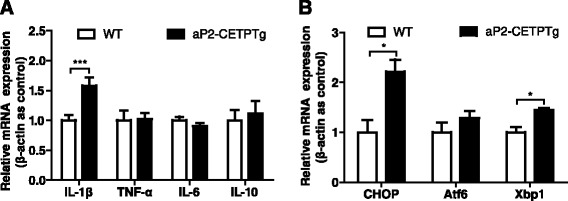
Expression of inflammation and ER stress related genes in isolated islets from aP2-CETPTg mice. **a** As for the mRNA level of pro-inflammatory markers, IL-1β was higher in aP2-CETPTg mice than their control, but TNF-α and IL-6 mRNA expression did not differ between the two groups. For the anti-inflammatory marker, IL-10 mRNA level had an increasing tendency in aP2-CETPTg mice. **b** Relative mRNA levels of CHOP and Xbp1 in isolated islets were increased in aP2-CETPTg mice. *n* = 4–6 per group. Values are expressed as mean ± SD. *p < 0.05, and *** *p* < 0.001 compared with control group. *Xbp1* X-box binding protein 1, *Atf6* activating transcription factor 6

Islet inflammation is emerging as an important factor that participates in the development of T2DM [21]. Thus, we sought to examine mRNA levels of IL-1β, TNF-α and IL-6, these representative pro-inflammatory cytokines that play key roles in modulating islet cytokine release and impairs islet function [21]. The results indicated that IL-1β mRNA was significantly increased in islets of aP2-CETPTg mice, but without differences in TNF-α, IL-6 or IL-10 mRNA expression (Fig. 9a).

Free cholesterol accumulation in macrophages leads to CHOP-induced ER stress [22]. To investigate whether unfolded protein response is enhanced in the islets from aP2-CETPTg mice, we measured the mRNA expression of CHOP, X-box binding protein 1 (Xbp1) and activating transcription factor 6 (Atf6). Our data showed that the mRNA levels of CHOP and Xbp1 were also increased in aP2-CETPTg mouse islets (Fig. 9b), suggesting that circulating CETP could be cytotoxic to beta cells. Atf6 did not differ between the islets from aP2-CETPTg and WT mice (Fig. 9b).

## Discussion

In this study, we demonstrated for the first time that circulating CETP, 1) exacerbate glucose intolerance, lower plasma insulin concentrations but increase insulin sensitivity; 2) increase free cholesterol accumulation in the islets; 3) reduce the expression of the genes which participate in beta cell proliferation and differentiation, resulting in fewer and smaller islet number and size; 4) induced the expression of the genes which are involved in inflammation, ER stress, and apoptosis; and 5) finally and most importantly, circulating CETP decreased insulin secretion from islet beta cells.

It is well known that insulin resistance and insulin deficiency are main factors of glucose intolerance. In our aP2-CETPTg mice, insulin sensitivity is increased, compared with WT mice. This could be due to two reasons, 1) adipocyte size becomes smaller [23], and 2) skeletal muscle takes up more glucose. Although aP2-CETPTg mice had increased insulin sensitivity, they displayed glucose intolerance and this could be due to beta cell dysfunction.

Circulating CETP influences insulin sensitivity and insulin secretion in a dose-dependent fashion. Recently, Raposo et al. [17] reported that CETP expression does not affect glucose homeostasis and insulin secretion. However, our GTT results clearly indicated that there is a CETP dose-dependent effect on mice glucose tolerance impairment and fasting insulin secretion dysfunction. Therefore, the inconsistent results between Raposo et al’s and ours’ could be mainly due to the different CETP levels in the blood. Raposo et al. utilized a heterozygous CETP transgenic (NFR-CETP-Tg) mice model which is identical to our NFR-CETP-Tg mice, presenting human like plasma levels of CETP (~2 ug/ml). However, our aP2-CETPTg mice have significantly higher levels of CETP in circulation. Therefore, elevated CETP mass and activity in T2DM patients may bring additional damage to insulin secretion.

What is the mechanism for beta cell dysfunction in aP2-CETPTg mice? We hypothesized that free cholesterol accumulation in aP2-CETPTg mice islet induce beta cell dysfunction. It is known that high cholesterol levels in islets interfere with glucose metabolism by inhibiting glucokinase activity, the critical glucose-sensing component of the beta cell, and reducing the effectiveness of the insulin secretory apparatus [4, 24]. ATP-binding cassette transporter A1 (ABCA1) is a cellular cholesterol transporter which participates in cholesterol efflux, mice lacking beta cell ABCA1 exhibited impaired glucose tolerance and decreased insulin secretion due to abnormalities of cholesterol homeostasis in isolated islets [25]. SREBP-2 is another member of the membrane-bound transcription factor SREBP family, and plays a crucial role in the regulation of cholesterol metabolism [19]. Expression of nuclear human SREBP-2 in beta cells resulted in severe diabetes due to defects in glucose- and potassium-stimulated insulin secretion. Cholesterol is a well-known component of both secretory granules and plasma membranes, and cholesterol-rich microdomains in the plasma membrane are thought to be essential for normal cell function, including exocytosis [26, 27]. The cholesterol-rich lipid rafts in beta cell membranes contain calcium channels and the proteins that constitute the secretory apparatus which consists of soluble N-ethylmaleimide-sensitive factor (NST) attachment protein receptor (SNARE), soluble NST attachment protein-25 (SNAP-25), and synaptobrevin. All of these proteins participate in insulin secretion. The accumulation of cholesterol in beta cell interferes with the effectiveness of the insulin secretory apparatus then decrease insulin secretion. Conversely, lowering beta cell cholesterol levels with methyl-β-cyclodextrin [4, 28], which depletes membrane cholesterol, increases insulin secretion. Our results indicated that FC/CE of islets was increased in the islets from aP2-CETPTg mice when compared with WT mice. Moreover, the free cholesterol accumulation is not directly result from ACAT mRNA level changes. Enrichment of FC changes beta cell membrane lipid composition and this could lead to less efficiency in insulin secretion through exocytosis.

Our study showed that the size of islets from aP2-CETPTg mice were significantly reduced, which could be another reason for the decrease of insulin secretion. These morphologic variations of islets in aP2-CETPTg mice might be a consequence of cholesterol accumulation in the beta cells. Studies in macrophages demonstrated that free cholesterol induces apoptosis by causing stiffening of the ER membrane, with subsequent induction of the unfolded protein response [29]. Our data also showed that beta cell apoptosis was increased, in line with higher expression of ER stress markers, including CHOP and Xbp1. Cholesterol accumulation can decrease expression of the beta cell specific proliferation and differentiation genes, Pdx1 and BETA2, which may be another reason for the decrease of beta cell number and size. Interestingly, Ki-67 mRNA expression was upregulated in aP2-CETPTg mice, which may be a feedback regulation of increased beta cell apoptosis. Islet inflammation has been reported in T2DM, and individuals with elevated serum levels of certain pro-inflammatory cytokines show increased risks for developing T2DM [30]. IL-1β is well established as a pro-inflammatory cytokine involved with the demise of the beta cell [31]. Our results showed that IL-1β mRNA was significantly increased in islets of aP2-CETPTg mice.

The question arises as to how CETP might promote free cholesterol accumulation in pancreatic beta cells. CETP has been reported to exchange lipids between cell membranes and between cell membranes and lipoproteins. For example, CETP can mediate efflux of cholesteryl ester from biological membranes to lipoproteins [32, 33]. CETP can also facilitate HDL-cholesterol selective uptake by adipocytes [34]. It is conceivable that CETP could also facilitate cholesteryl ester efflux out of the plasma membrane of cell, thus causing free cholesterol accumulation in the cells. It has been suggested that CETP could enhance the exchange of lipids during the formation of a ternary collision complex consisting of donor and acceptor lipoproteins, bridged by CETP [35]. By analogy, CETP might bind the HDL to the plasma membrane of cell, and increase the collisions between the membrane and the lipoprotein, allowing lipids to be exchanged in the process. We speculate that circulating CETP could transfer cholesteryl ester but not free cholesterol out of the beta cell to lipoproteins in the circulation, resulting in elevation of FC/CE ratio in the islets, and the precise mechanism needs further study.

## Conclusions

In summary, our data show that circulating CETP have an impact on beta cell cholesterol homeostasis by accumulation of free cholesterol, thus reducing beta cell insulin secretion. These data provide further evidence to the concept that regulation of beta cell cholesterol homeostasis is essential for normal islet function. CETP inhibition could be a potential promising approach to improve beta cell function in T2DM by decreasing islet cholesterol accumulation and inflammation.

### Ethics approval

All animal experiments were conducted in accord with accepted standards of humane animal care, as outlined in the Ethical Guidelines, and were approved by the Research Animal Care Committee of Nanjing Medical University (IACUC-14030136).

### Consent for publication

Not applicable.

## Abbreviations

ABCA1: ATP-binding cassette transporter A1
Atf6: activating transcription factor 6
aP2-CETPTg: adipose tissue-specific CETP expression transgenic
CE: cholesteryl esters
CETP: cholesteryl ester transfer protein
FC: free cholesterol
GSIS: glucose-stimulated insulin secretion
HDL: high density lipoprotein
HMGCS: 3-hydroxy-3-methylglutaryl-CoA synthase
IPGTT: intraperitoneal glucose tolerance test
LDL: low density lipoprotein
LDLR: LDL receptor
Pdx1: pancreatic and duodenal homeobox 1
SNAP: soluble N-ethylmaleimide-sensitive factor attachment protein
SNARE: soluble N-ethylmaleimide-sensitive factor attachment protein receptor
SREBP-2: sterol regulatory element-binding protein-2
T2DM: type 2 diabetes mellitus
TUNEL: transferase-mediated dUTP nick-end labeling
VLDL: very low density lipoprotein
WT: wild type
Xbp1: X-box binding protein 1

## References

[1] Cerf ME (2013). Beta cell dysfunction and insulin resistance. Front Endocrinol (Lausanne).

[2] Prentki M, Nolan CJ (2006). Islet beta cell failure in type 2 diabetes. J Clin Invest.

[3] Poitout V, Robertson RP (2008). Glucolipotoxicity: fuel excess and beta-cell dysfunction. Endocr Rev.

[4] Hao M, Head WS, Gunawardana SC, Hasty AH, Piston DW (2007). Direct effect of cholesterol on insulin secretion: a novel mechanism for pancreatic beta-cell dysfunction. Diabetes.

[5] Fryirs M, Barter PJ, Rye KA (2009). Cholesterol metabolism and pancreatic beta-cell function. Curr Opin Lipidol.

[6] Yamashita S, Hirano K, Sakai N, Matsuzawa Y (2000). Molecular biology and pathophysiological aspects of plasma cholesteryl ester transfer protein. Biochim Biophys Acta.

[7] Jiang XC, Moulin P, Quinet E, Goldberg IJ, Yacoub LK, Agellon LB, Compton D, Schnitzer-Polokoff R, Tall AR (1991). Mammalian adipose tissue and muscle are major sources of lipid transfer protein mRNA. J Biol Chem..

[8] Barter PJ, Rye KA, Tardif JC, Waters DD, Boekholdt SM, Breazna A, Kastelein JJ (2011). Effect of torcetrapib on glucose, insulin, and hemoglobin A1c in subjects in the Investigation of Lipid Level Management to Understand its Impact in Atherosclerotic Events (ILLUMINATE) trial. Circulation.

[9] Siebel AL, Natoli AK, Yap FY, Carey AL, Reddy-Luthmoodoo M, Sviridov D, Weber CI, Meneses-Lorente G, Maugeais C, Forbes JM, Kingwell BA (2013). Effects of high-density lipoprotein elevation with cholesteryl ester transfer protein inhibition on insulin secretion. Circ Res..

[10] de Vries R, Perton FG, Dallinga-Thie GM, van Roon AM, Wolffenbuttel BH, van Tol A, Dullaart RP (2005). Plasma cholesteryl ester transfer is a determinant of intima-media thickness in type 2 diabetic and nondiabetic subjects: role of CETP and triglycerides. Diabetes..

[11] Smaoui M, Hammami S, Attia N, Chaaba R, Abid N, Kilani N, Kchaou H, Mahjoub S, Abid M, Hammami M (2006). Modulation of plasma cholesteryl ester transfer protein activity by unsaturated fatty acids in Tunisian type 2 diabetic women. Nutr Metab Cardiovasc Dis..

[12] Dallinga-Thie GM, Dullaart RP, van Tol A (2007). Concerted actions of cholesteryl ester transfer protein and phospholipid transfer protein in type 2 diabetes: effects of apolipoproteins. Curr Opin Lipidol.

[13] MacLean PS, Vadlamudi S, MacDonald KG, Pories WJ, Barakat HA (2005). Suppression of hepatic cholesteryl ester transfer protein expression in obese humans with the development of type 2 diabetes mellitus. J Clin Endocrinol Metab.

[14] Ju X, Cui Q, Zhang M, Wang W, Jiang J, Chang Y, Wang K, Yang T, Zhou H. Human cholesteryl ester transfer protein enhances insulin-mediated glucose uptake in adipocytes. Life Sci. 2011;89:479–84. 10.1016/j.lfs.2011.07.014 21816162

[15] Zhou H, Li Z, Hojjati MR, Jang D, Beyer TP, Cao G, Tall AR, Jiang XC (2006). Adipose tissue-specific CETP expression in mice: impact on plasma lipoprotein metabolism. J Lipid Res..

[16] Jiang XC, Agellon LB, Walsh A, Breslow JL, Tall A (1992). Dietary cholesterol increases transcription of the human cholesteryl ester transfer protein gene in transgenic mice. Dependence on natural flanking sequences. J Clin Invest.

[17] Raposo HF, Vanzela EC, Berti JA, Oliveira HC (2016). Cholesteryl Ester Transfer Protein (CETP) expression does not affect glucose homeostasis and insulin secretion: studies in human CETP transgenic mice. Lipids Health Dis.

[18] Zhu SS, Ren Y, Zhang M, Cao JQ, Yang Q, Li XY, Bai H, Jiang L, Jiang Q, He ZG, Chen Q (2011). Wld(S) protects against peripheral neuropathy and retinopathy in an experimental model of diabetes in mice. Diabetologia..

[19] Ishikawa M, Iwasaki Y, Yatoh S, Kato T, Kumadaki S, Inoue N, Yamamoto T, Matsuzaka T, Nakagawa Y, Yahagi N (2008). Cholesterol accumulation and diabetes in pancreatic beta-cell-specific SREBP-2 transgenic mice: a new model for lipotoxicity. J Lipid Res.

[20] Rumsey SC, Galeano NF, Lipschitz B, Deckelbaum RJ (1995). Oleate and other long chain fatty acids stimulate low density lipoprotein receptor activity by enhancing acyl coenzyme A:cholesterol acyltransferase activity and altering intracellular regulatory cholesterol pools in cultured cells. J Biol Chem.

[21] Kashiwagi A, Takahashi H, Ishikawa H, Yoshida S, Kazuta K, Utsuno A, Ueyama E (2015). A randomized, double-blind, placebo-controlled study on long-term efficacy and safety of ipragliflozin treatment in patients with type 2 diabetes mellitus and renal impairment: results of the long-term ASP1941 safety evaluation in patients with type 2 diabetes with renal impairment (LANTERN) study. Diabetes Obes Metab..

[22] Feng B, Yao PM, Li Y, Devlin CM, Zhang D, Harding HP, Sweeney M, Rong JX, Kuriakose G, Fisher EA (2003). The endoplasmic reticulum is the site of cholesterol-induced cytotoxicity in macrophages. Nat Cell Biol..

[23] Zhou H, Li Z, Silver DL, Jiang XC (2006). Cholesteryl ester transfer protein (CETP) expression enhances HDL cholesteryl ester liver delivery, which is independent of scavenger receptor BI, LDL receptor related protein and possibly LDL receptor. Biochim Biophys Acta.

[24] Vikman J, Jimenez-Feltstrom J, Nyman P, Thelin J, Eliasson L (2009). Insulin secretion is highly sensitive to desorption of plasma membrane cholesterol. FASEB J.

[25] Brunham LR, Kruit JK, Pape TD, Timmins JM, Reuwer AQ, Vasanji Z, Marsh BJ, Rodrigues B, Johnson JD, Parks JS (2007). Beta-cell ABCA1 influences insulin secretion, glucose homeostasis and response to thiazolidinedione treatment. Nat Med..

[26] Salaun C, James DJ, Chamberlain LH (2004). Lipid rafts and the regulation of exocytosis. Traffic.

[27] Wang Y, Thiele C, Huttner WB (2000). Cholesterol is required for the formation of regulated and constitutive secretory vesicles from the trans-Golgi network. Traffic.

[28] Xia F, Gao X, Kwan E, Lam PP, Chan L, Sy K, Sheu L, Wheeler MB, Gaisano HY, Tsushima RG (2004). Disruption of pancreatic beta-cell lipid rafts modifies Kv2.1 channel gating and insulin exocytosis. J Biol Chem.

[29] Yao PM, Tabas I (2001). Free cholesterol loading of macrophages is associated with widespread mitochondrial dysfunction and activation of the mitochondrial apoptosis pathway. J Biol Chem.

[30] Spranger J, Kroke A, Mohlig M, Hoffmann K, Bergmann MM, Ristow M, Boeing H, Pfeiffer AF (2003). Inflammatory cytokines and the risk to develop type 2 diabetes: results of the prospective population-based European Prospective Investigation into Cancer and Nutrition (EPIC)-Potsdam Study. Diabetes..

[31] Dinarello CA, Donath MY, Mandrup-Poulsen T (2010). Role of IL-1beta in type 2 diabetes. Curr Opin Endocrinol Diabetes Obes.

[32] Stein O, Halperin G, Stein Y (1986). Cholesteryl ester efflux from extracellular and cellular elements of the arterial wall. Model systems in culture with cholesteryl linoleyl ether. Arteriosclerosis.

[33] Stein Y, Stein O, Olivecrona T, Halperin G (1985). Putative role of cholesteryl ester transfer protein in removal of cholesteryl ester from vascular interstitium, studied in a model system in cell culture. Biochim Biophys Acta.

[34] Vassiliou G, McPherson R (2004). Role of cholesteryl ester transfer protein in selective uptake of high density lipoprotein cholesteryl esters by adipocytes. J Lipid Res.

[35] Tall A (1995). Plasma lipid transfer proteins. Annu Rev Biochem.

